# Harvard Odontological Society

**Published:** 1894-06

**Authors:** 

**Affiliations:** Harvard Odontological Society


### HARVARD ODONTOLOGICAL SOCIETY.

A MONTHLY meeting of the Harvard Odontological Society was held Thursday evening, December 28, 1893, at Young's Hotel, Boston, Dr. Eddy, President, in the chair.

*President Eddy.*—The subject of "Porcelain Contours," Dr. Grant's paper, is now open for discussion.

(For this paper, see page 309.)

DISCUSSION.

*Dr. Cooke.*—I would like to ask Dr. Grant if all these wheels which he passed around to-night were made by the same firm in Waltham?

*Dr. Grant.*—They were. Two of these wheels, in the form of saws, are made for cutting off teeth, and they do the cutting very smoothly indeed. There is none of that rasping or jumping that you get from a saw. The gentlemen seem to be accommodating and anxious to make good instruments.

*Dr. Cooke.*—What cement do you use?

*Dr. Grant.*—I have used Caulk's for a long time. It is not as satisfactory as I would like, but it is the best cement I know of.

*Dr. Cooke.*—White or yellow?

*Dr. Grant.*—I feel a little safer when I use the gray.

*Dr. Cooke.*—There was one more point that I did not get clearly. What do you use on the wheel?

*Dr. Grant.*—Alcohol and carbolic acid.

*Dr. Cooke.*—What proportion?

*Dr. Grant.*—About ten to fifteen per cent. of carbolic acid.

*Dr. Giblin.*—Will Dr. Grant please tell us if he would feel confident in fastening the dowel-posts with jewellers' enamel and blowpipe? It is so difficult to use the furnace that one does not care to do it any oftener than he is actually obliged to.

*Dr. Grant.*—I would say that I have done that with large pieces, but I feel a little safer if it is baked in a furnace, on account of the more even, steady heat. I have fastened metallic pins into pivot teeth with jewellers' enamel, and also with glass, in the way you speak of, and they held securely; but it is risky to try on a tip that you have spent a good deal of time over. The blow-pipe is apt to discolor except with a perfectly blue flame. One is more sure of the color if he gets even heat.

*Dr. Werner.*—How does this method compare for time? Do you think you save time, generally speaking, compared with gold contours?

*Dr. Grant.*—I don't know that I should save time. In the average contouring of a central I should expect to spend from two and a half to four hours.

*Dr. Werner.*—I have never contoured with porcelain except in one instance. My assistant did the work of fitting the piece to be put on. I found he was not very enthusiastic over it, and I think it is very difficult work, too much to turn over to anybody, and yet it seems to be desirable in many cases until we find that ideal filling for which we have been waiting so long.

*Dr. Grant.*—I don't know why I should be any more willing to turn over such a piece of work as that to an assistant than I should a gold filling. One is quite as important as the other, and if you are going to turn such work over to an assistant, you may as well turn it all over to assistants and take a vacation yourself. I don't like to hear a man urge the difficulty of an operation as an argument against it. We, as dentists, pride ourselves on—and one of the highest claims of dentistry is—the skill in the manipulation that its exponents exhibit. Wherever a new thing is introduced I hear two objections,—first, it takes a great deal of time; secondly, it is hard to do. Now, we should not look at it from that point of view. Every good thing is difficult to do, it does not matter what it is, and the longer I live the more I am convinced of that fact; and as skilful men, which we claim to be, we should not let such considerations deter us from adopting anything which is going to prove beneficial.

*Dr. Hitchcock.*—I would like to ask Dr. Grant if he ever screws on his tips?

*Dr. Grant.*—You mean screw the dowel into the tooth and then set the tip on?

*Dr. Hitchcock.*—Yes.

*Dr. Grant.*—It can be done, but I have found it seldom possible to make such a small thread hold satisfactorily in tooth substance; and besides that, as I said before, there is a great advantage in vitrifying the cement which holds the dowel in the tooth. If set in phosphate, you will be very likely to see the dowel, but if it is baked in there you do not see it any more than you see the pins in a porcelain tooth.

*Dr. Hitchcock.*—I have seen threads hold so firmly that it was almost impossible to get them out.

*Dr. Grant.*—Well, they will not pull out, but they will work out after a time. I have set screws in pivot teeth, and they would come to me so loose that I expected to remove them with my fingers, but I found they did not come out so easily. There is the objection, however, that there is not toughness enough in dentine to properly hold the thread.

*Dr. Giblin.*—Do you serrate the portion of the dowel-post that goes into the tooth?

*Dr. Grant.*—Yes, a little.

*President Eddy.*—I should like to hear a few words from our guest, Dr. Church, on this subject. I know he has had considerable experience in this line.

*Dr. Church.*—Mr. President and Gentlemen of the Odontological Society, I really have not much to add to Dr. Grant's experience; it has been longer than mine. I agree with many of his points.

Though I have never followed out his ideas exactly, I have used the principle a good deal. He is the first one I have heard express the absolute necessity for a perfectly flat, lapped joint for the porcelain tooth to fit against. I have worked many a long hour to get at that perfect color, and have sometimes been almost discouraged on account of the long, laborious work it compels. I have worked somewhat after the Land process, carving the piece on. I do not reach that fine blending of color that I would like, but the results in many ways are such an improvement over, at least, the gold filling, that I consider it ample justification for what I do. I have used porcelain in making inlays to be put over abraded surfaces. It requires a great amount of skill and a very careful adjustment of shade, and I find, as time goes on and I grow more critical of my own work, that the labor does not seem to lessen at all. But even the results that I have reached seem to me infinitely better than adorning a man with the sign-board of a big gold filling on the face of the tooth, and I think that is a point which you are all beginning to appreciate. I have also used it in other ways with a good deal of satisfaction. Take, for instance, a very weak tooth, where one point is abraded and there are cavities, both mesial and distal. Where there is a sufficient material, I have made inlays to fit both cavities, strengthening the weak point, and those teeth have exceeded my expectations. I have one case where several porcelain inlays have been in one mouth for a year and a half now, and not one has yet failed. I think it is a great blessing to humanity to have some sort of riddance from such ornamental things as large gold fillings.

Henry L. Upham, D.M.D.,
*Editor Harvard Odontological Society.*

